# Artificial intelligence and the aging mind

**DOI:** 10.18632/aging.204644

**Published:** 2023-04-03

**Authors:** Jeyeon Lee, Leland R. Barnard, David T. Jones

**Affiliations:** 1 Department of Radiology, Mayo Clinic, Rochester, MN 55905, USA; 2 Department of Neurology, Mayo Clinic; Rochester, MN 55905, USA

**Keywords:** brain age, artificial intelligence, Alzheimer’s dementia, neurodegenerative disease, biomarker

Aging is a significant risk factor for many diseases, including Alzheimer’s dementia (AD), but our understanding of the physiology of aging and its relationship to AD and related disorders (ADRD) is incomplete. Artificial intelligence (AI) and machine learning (ML) technologies can help us better understand these diseases and aging itself by using biological data from the brain or other sources to create a mapping between age and biological data. These mappings can be used to train AI/ML models to predict age, which is then interpreted as representing a biological age or “physiological clock” that may not necessarily match the individual’s chronological age [1]. The gap between the biological and chronological age can be used as a biomarker for age-related diseases that manifest in the biological data being used in the model. When the data being used is related to the brain, then this difference is referred to as a brain age gap. In medicine, it is well known that intrinsic factors (e.g., genes), extrinsic factors (environment), growth and other developmental factors also contribute to variation around normal aging patterns [2]. This variation is indexed, along with disease related variation, by any measure of deviance from normal aging trajectories [3].

Recently, we developed convolutional neural network-based brain age prediction models using a large collection (2,349 unique individuals with 4,127 scans) of brain magnetic resonance imaging (MRI; structural image) and brain fluorodeoxyglucose positron-emission tomography (FDG-PET; metabolic image) in people aged from 26 to 98 years old [4]. In a sample of cognitively normal individuals (n=1,805), the AI models showed accurate brain age estimation of which a mean absolute error (MAE; unit, years) was 3.08±0.14 and 3.49±0.16 for FDG- and MRI-based model, respectively. *Post-hoc* interpretability analysis explaining which brain regions contribute most to age prediction revealed modality-specific aging patterns. Metabolic brain aging was characterized by a transition of posterior-to-anterior structures with increased age, which coincided with previous functional MRI findings. In contrast, a higher contribution of cerebral spinal fluid (CSF) boundaries was dominantly found in the MRI model, which may reflect the typical enlargement of the CSF spaces as brain ages. Despite the distinct patterns, both models’ predictions yielded a high correlation suggesting shared sources of age-related variability between the metabolic and structural changes. In addition, higher brain age gaps were estimated in cohorts with neurodegenerative disorders, including mild cognitive impairment (MCI), AD, frontotemporal dementia (FTD), and dementia with Lewy bodies (DLB) than normal controls. The brain age gap was strongly associated with pathologic tau protein levels and cognitive test scores and showed longitudinal predictive ability for cognitive decline in ADRD, including normal control individuals who converted to MCI. Interestingly, the brain imaging patterns generating brain age gaps in AD showed higher similarity with normal aging than other neurodegenerative syndromes implying that AD might be more like an accelerated representation of biological aging than others.

These AI/ML technologies for studying brain aging require large amounts of high-quality biologically relevant data to serve as the ‘language’ for this mapping. Using different data would be analogous to using a different language to describe the relationship between aging and dementia. A major challenge in using these technologies is that we do not always understand the semantics of the language that they are written in, and we must use associations with existing knowledge as a sort of Rosetta stone to decode them. That is why any interpretation of these models should be firmly grounded in other biological markers of the disease process of interest and a general biology of aging. To date, brain age research is dominated by group-wise statistics due to high variability found in the individual subject level. This hampers a successful translation of the brain age gap to clinical practice, although it has proven to be a meaningful imaging biomarker for clinical factors. State-of-the-art neuroimaging-based brain age prediction achieved a MAE of 2.14 years [5]. Advances of AI/ML technique and growth in data availability could further increase the performance of prediction; however, clinically meaningful reductions in MAE may not be realistic given the high variability of real-world data [6]. The sources of variability include not only technical variance, such as scanner, preprocessing, etc., but also biological variance due to complex physiological processes relevant to aging, sex, ethnicity, life style and other intrinsic and extrinsic factors [3, 7].

Although more research and optimization are needed to determine its clinical usefulness, the study of brain age has great potential as a tool for understanding brain aging and age-related diseases. The pathologic condition and changes due to biological aging often are not easily dissociable because aging entails cumulative biological damage and allostatic responses against that damaging perturbation over time [3]. Our study found that with greater severity in the disease states such as AD, FTD, and DLB, the brain imaging patterns associated with the brain age gap was more pathology-specific than that of MCI. In this conceptual framework, brain age research can further facilitate our understanding of the relationship between normal biological aging and pathologically-driven aging in a broad sense. The brain age gap may also be useful as a prognostic biomarker, as our results showed that individuals with higher brain age gaps at baseline were more likely to experience disease progression, even in the preclinical stage. The fact that the brain age gap is a comprehensive and intuitive measure of disease severity using biological data that is already being acquired in clinical practice, makes it an attractive biomarker for further development for clinical use [8].
